# Determinants of human papillomavirus vaccine attitudes: an interview of Wisconsin parents

**DOI:** 10.1186/s12889-018-5635-y

**Published:** 2018-06-15

**Authors:** Kathrine L. Barnes, Jeffrey J. VanWormer, Shannon Stokley, Elizabeth R. Vickers, Huong Q. McLean, Edward A. Belongia, Casper G. Bendixsen

**Affiliations:** 1 National Farm Medicine Center, Marshfield Clinic Research Institute, 1000 North Oak Ave, Marshfield, WI 54449 USA; 2 U.S. Centers for Disease Control and Prevention, Atlanta, USA

**Keywords:** Human papillomavirus vaccine, Attitudes, Interviews, Parents

## Abstract

**Background:**

Parental attitudes play a key role in their decisions to vaccinate adolescents against the human papillomavirus (HPV). Little is known, however, about the formative experiences that shape parents’ attitudes about the HPV vaccine.

**Methods:**

We conducted semi-structured interviews with 25 parents of 11–17 year old adolescents in Wisconsin who changed their HPV vaccine attitudes (per prior surveys) over one year. A modified grounded theory approach was then used to generate primary themes of attitudinal determinants.

**Results:**

Participants were predominately mothers. We identified three major themes that shaped parents’ HPV attitudes: (1) the perceived likelihood of the HPV vaccine preventing cancer, (2) agency in adolescence and gauging their adolescent child’s intent for sexual activity, (3) the credibility of HPV vaccine information sources. General messaging around cancer prevention did not always supersede some parents’ concerns about the vaccine’s perceived link to sexual activity. Parents often viewed their adolescent child’s feelings about the HPV vaccine as a gauge of their (child’s) intent for sexual activity. Interviewees felt a sense of responsibility to educate themselves about the HPV vaccine using multiple sources and particularly looked to their medical provider to filter conflicting information.

**Conclusions:**

More family-specific (vs. disease-prevention) messaging and recommendations may be needed in the clinical environment to sway some parents’ negative attitudes about the HPV vaccine. Future research should explore additional strategies to improve HPV vaccine attitudes, such as situating the vaccine in the context of a monogamous lifestyle that many parents wish to impart to their children.

## Background

One in four Americans is currently infected with human papillomavirus (HPV), with 14 million new cases each year [1]. Most HPV infections resolve on their own, but some infections can lead to cancers, such as cervical and oropharyngeal cancers. The HPV vaccine was introduced in the United States in 2006 and subsequently recommended for both girls and boys age 11–12 years to prevent cancers, genital warts, and other HPV-related conditions [2]. Complete HPV vaccine coverage could prevent more than 90% of all HPV-related cancers [3]. Since the vaccine is recommended in early adolescence, and before the onset of sexual activity, parents’ acceptance of the HPV vaccine is believed to be a crucial factor in ultimately increasing coverage rates [4]. Although HPV vaccination rates have increased, they remain much lower than rates for other adolescent vaccines [5].

Prior research suggests parent acceptability of the HPV vaccine is largely driven by their attitudes and beliefs regarding the vaccine’s effectiveness, safety, and ease of access [6–9]. Although parental attitudes may be a modifiable factor, most research on this topic has relied on cross-sectional surveys focused on general beliefs about the HPV vaccine’s propensity to cause harm or deliver benefits, with some studies examining sociodemographic predictors of HPV vaccine hesitancy [10, 11]. In-depth interviews with parents are less common and typically cover general awareness and knowledge of HPV as part of broader topics about preventing sexually transmitted infections [12]. One interview study found that parents routinely delay their decisions to vaccinate against HPV based on beliefs that their adolescent children are at low risk for acquiring HPV infection [13].

The specific social, environmental, and/or clinical experiences that are most influential in shaping parents’ interpersonal acceptance (or rejection) of the HPV vaccine remain unclear. To better understand how prior experiences influence attitudinal formation, shifts, and decision-making about the HPV vaccine, we conducted a qualitative study using in-depth interviews of mothers and fathers of adolescents whose attitudes about HPV vaccination changed over the prior year. Elucidating HPV vaccine attitude antecedents could generate directions for future research on HPV vaccine education and promotion, particularly within clinical settings where preventive health discussions are common.

## Methods

### Design and setting

This was a qualitative study that used semi-structured interviews with parents of adolescents who were eligible for the HPV vaccine. It was conducted within the Marshfield Clinic Health System (MCHS), a large integrated care system in north-central Wisconsin. This region is predominately rural. Parent interview activities were conducted as part of a larger study designed to increase HPV vaccine coverage in MCHS clinics (described further elsewhere [14]). As part of the broader study, a baseline and 1-year follow-up survey of HPV vaccine attitudes was administered to 221 parents of 11–17 year old adolescent MCHS patients who had not completed the HPV vaccine series at baseline and who were homed to one of the three largest MCHS centers targeted in the main study (Marshfield, Wausau/Weston, Eau Claire/Chippewa Falls). Parents completed the Carolina HPV Immunization Attitudes and Beliefs Scale (CHIAS) survey [15] to assess how changes in their CHIAS attitudinal scores for barriers, harms, ineffectiveness, and uncertainties influenced whether or not their adolescent children received the HPV vaccine. Possible scores for each area range from 1 to 4 points, with lower scores indicating more favorable HPV vaccine attitudes. Findings indicated that decreased parental uncertainties score over one year was a significant predictor of vaccine receipt in their adolescent children. Also, greater harms score at baseline was a significant predictor of lower HPV vaccine series completion [16].

### Participants

Participants lacking conversational competency in English were excluded. Inclusion criteria for interview participants were: (1) completed the baseline and 1-year follow-up CHIAS surveys administered in spring 2015 and spring 2016, and (2) experienced an attitudinal change, either more or less favorable, in at least one of the four CHIAS scores for perceived barriers, harms, ineffectiveness, or uncertainties. Attitudinal change was defined as scoring below the 20th percentile or above the 80th percentile of change, as measured between baseline and follow-up surveys, on at least one CHIAS score [15]. As an example, a parent would be study-eligible if they were below the 20th percentile in the distribution of CHIAS barriers change scores, which would reflect a lower CHIAS barriers score at follow-up relative to baseline (i.e., a more favorable change in attitudes pertaining to HPV vaccine barriers). Similarly, a parent would also be study-eligible if they were above the 80th percentile in the distribution of CHIAS harms change scores, which would reflect a higher CHIAS harms score at follow-up relative to baseline (i.e., a less favorable shift in attitudes pertaining to HPV vaccine harms). In contrast, a parent would not be study-eligible if there was no change in any CHIAS scores, as they would be at or near the 50th percentile (median) in the distribution of CHIAS change scores. Per the original survey study eligibility criteria described previously, adolescents did not have a completed HPV vaccine series at the time of their baseline survey, but they may or may not have gone on to initiate or complete the HPV vaccine series by the time of these interviews with their parents.

### Recruitment

All eligible participants (regardless of the direction of attitudinal change) were randomly selected for interviews, with an initial goal of 30 enrollees, or fewer if saturation in interviewee themes was reached. Recruitment was conducted over approximately two months beginning in late summer 2016. Potential participants were contacted by telephone up to four times to schedule an interview. Interviews were completed approximately six months after participants completed their follow-up attitudinal survey (~ 18 months after they completed their baseline attitudinal survey). Study procedures were approved by the Marshfield Clinic Institutional Review Board, and all participants provided informed consent. Participants received a $20 gift card upon completion of their study interview.

### Data collection

Study interviews were performed in a private non-clinical space, such as a conference room or empty office. All interviews were conducted by the same interviewer (KB), who is a trained ethnographic researcher and is experienced in qualitative interviewing techniques. Interviews were designed to last approximately one hour and were digitally recorded. The interview guide was first developed by the interviewer and refined with input from the study team. Parents were queried about their first impressions of the HPV vaccine, including the perceived risks and benefits, related clinical encounters, and their child’s perceived HPV vaccination status (the interviewer did not review adolescent vaccination records prior to the interview), as well as their views on child autonomy, parental decision-making, and how said views have changed over time as they relate to HPV vaccination. As described in further detail by Bernard [17], no education on adolescent vaccine clinical guidelines was proffered as part of the interviews, but the anthropologic interviewing method used in this study did not position the interviewer as a completely passive observer. It instead attempted to engage the interviewee in more conversational tones and also permitted the use of targeted probes to collect deeper knowledge into participants’ reasoning, or lived experience, as related to their HPV vaccine attitudes. Interview data was linked to the participants’ adolescent children’s stored Marshfield Clinic medical records, including prior vaccination history for recommended adolescent vaccines. Interviewees’ prior survey responses on sociodemographic factors were also included for descriptive purposes.

### Analysis

Digitally recorded interviews were transcribed into query-able text using a professional transcription service (GMR Transcription Services, Tustin, CA) and analyzed using NVIVO qualitative data analysis software (QSR International, Melbourne, Australia). Similar to Hughes, et al. [13] and detailed further by Kelle [18], analyses were based on a modified grounded theory approach. Seven deductive codes were provided to three coders on the study team (KB, CB, EV). These codes were generated by the interviewer based on the interview items and prior survey results. One open coding category was generated through inductive coding and coder consensus. Analyses occurred after transcription of the interviews was complete. Interviews were stratified by length and randomly assigned to either a trained ethnographer (CB, *n* = 13) or an epidemiologist specializing in vaccine studies (EV, *n* = 12). The interviewer (KB) randomly selected half of each person’s transcripts to code a second time. Coders met regularly to discuss common themes. A final meeting was held with a broader team of study researchers, with themes being finalized based on the consensus of the coders with input from the full research team. It was realized that parents could be categorized broadly into those who vaccinate their children against HPV or not, as parents who vaccinated their kids (most of our sample) consistently reiterated public health messaging about the importance of HPV vaccination for cancer prevention. Parents who did not vaccinate their children against HPV, however, were clearly less persuaded by such public health messages. Thus our thematic conclusions, while covering both parent categories, focused somewhat more on contrasts drawn from parents with less favorable HPV vaccine attitudes as a more likely target of future (refined) educational messaging or clinical interventions.

## Results

Of the 221 original baseline survey respondents, there were 140 who were eligible for this interview study because they also returned a 1-year follow-up survey and experienced an attitudinal change. Interview recruitment was truncated after 25 participants were enrolled, as theme saturation was achieved. Interviews lasted between 30 and 90 min. As outlined in Table 1, participants were predominantly female and ranged in age from 37 to 63 years. They were also typically college-educated, married or partnered, and employed in education or healthcare sectors. Per study eligibility criteria, none of the parents had an adolescent child who completed the HPV vaccine series at the time of the baseline survey. Twenty eight percent of parents’ adolescent children had received at least one HPV vaccine dose at baseline and, by the time of their interview, 72% of their adolescent children had gotten at least one HPV vaccine dose (and 40% had completed the 3-dose vaccine series). By comparison, 84% received the Tdap and meningococcal conjugate vaccine and 56% of their adolescent children received the prior season (2015–2016) influenza vaccine (at the time of their interview). Comparisons across these common adolescent vaccines are also highlighted further below, where relevant, to help contextualize attitudes about the HPV vaccine in particular.

**Table 1:** Characteristics of Parents interviewed about their Attitudes and Beliefs regarding the HPV Vaccine

	*N* = 25
Parents’ age (years)	
< 40	20%
41–49	64%
≥ 50	16%
Age of parents’ adolescents (years)	
11–12	56%
13–14	24%
≥ 15	20%
Gender	
Female	84%
Male	16%
Race/ethnicity	
White, Non-Hispanic	96%
Not White or Hispanic	4%
Community	
Marshfield	13%
Wausau / Weston	52%
Eau Claire / Chippewa Falls	20%
Education	
High school or less	4%
Some college	16%
Associates degree	24%
Bachelor’s degree	32%
Graduate degree	24%
Health insurance	
Private	84%
Public-assisted	16%
Marital status	
Married / partnered	92%
Not Married / partnered	8%
Employment sector	
Education	36%
Healthcare	28%
Business	16%
Other	8%
Not employed	12%

Values are reported as % of column total

CHIAS = Carolina HPV Immunization Attitudes and Beliefs Scale

Per study eligibility criteria, all interviewed parents experienced a relative shift in at least one CHIAS score during the year between the baseline and follow-up attitudinal surveys. But as outlined in Table 2, across all interviewed parents, mean CHIAS scores either did not change or shifted toward a slightly more favorable HPV vaccine attitude score at follow-up. Only the CHIAS score for ineffectiveness showed a statistically significant decrease by 0.5 points (~ 25% improvement; *p* = 0.013). Major absolute changes in attitude scores were uncommon and no interviewed parent experienced a complete reversal of attitudinal direction between the two survey time points (i.e., from completely supportive of the HPV vaccine to unsupportive, or vice versa). Thus the ensuing themes reflected determinants of parents’ current HPV vaccine views, or recent subtle evolutions in said attitudes. When queried about changes in their HPV vaccine attitudes, most participants responded that they have been stable for some time. For example, one parent responded, “I would say very consistent… we haven’t really wavered [on vaccinations and immunizations in general] and always made sure our kids are up to date.” Another parent described researching the HPV vaccine and reflected, “right from the start when I kind of started looking into it I just kind of thought, well, I don’t think this is one that my child’s gonna get.”

**Table 2:** Baseline, 1-year Follow-up, Change (including relevant percentile ranks) in CHIAS Scores among North-Central Wisconsin Parents (*N* = 25)

CHIAS scores (1–4 points)	Baseline	Follow-up	Change (follow-up minus baseline points)
Barriers	1.3 ± 0.4	1.2 ± 0.3	−0.1 ± 0.4
< 20th percentile rank			28%
> 80th percentile rank			16%
Harms	2.0 ± 0.7	2.0 ± 0.6	0.0 ± 0.4
< 20th percentile			20%
> 80th percentile			20%
Uncertainties	1.8 ± 0.6	1.7 ± 0.6	− 0.1 ± 0.6
< 20th percentile			24%
> 80th percentile			24%
Ineffectiveness	1.9 ± 0.9	1.4 ± 1.0	−0.5 ± 0.9
< 20th percentile			36%
> 80th percentile			16%

Values are reported as mean ± SD or percent of sample

Three themes specific to HPV vaccination emerged from the study interviews (see Table 3 for more example quotes): (1) the perceived likelihood of the HPV vaccine preventing cancer, (2) agency and adolescence and gauging their adolescent child’s intent for sexual activity, (3) the credibility of HPV vaccine information sources.

**Table 3:** Interview themes and example quotes

Parent, Vaccination History of adolescent child	Quote
Theme 1: Perceived likelihood of the HPV vaccine preventing cancer	
Parent, age 36 Adolescent, age 13 Vaccinations: Tdap Meningococcal	*[Cancer not motivating]* So when I looked up the research on it, it was kind of one of my things was okay, I understand where they’re [physicians are] coming from, but there’s over 100 strains of HPV and this is only protecting against four. There’s the one company that they say it’s like six or seven. But out of all those strains, and the four that they’re covering, only two of those strains of HPV cause cervical cancer. And yes they cause like 70% of all forms of cervical cancer. Yet out of all forms of cancer, cervical cancer is the most treatable. As long as you’re getting your yearly pap smears, you’re gonna catch it.
Parent, age 55 Adolescent, age 12 Vaccinations: HPV (complete) Tdap Meningococcal	*[Cancer as motivating]* Just knowing about like what can happen. It could be terminal. It’s just like our fears. Like, parents say whether they’d be more apt to engage in sex – it’s up to me – it’s a lack of trust in your child – your own fears. And obviously, working here in the hospital and seeing people with all sorts of different cancers.
Parent, age 50 Adolescent, age 14 Vaccinations: 1–2 HPV shots, did not complete by follow-up Tdap Meningococcal Influenza	*[Cancer as motivating]* Yeah, and I think the association seems to be maybe strongest for that, and maybe the preventive is even stronger too, or the effectiveness, but it’s still very strong for some of the male cancers. And then also, you don’t want to be responsible for transmitting either, so there’s that as well. So I can see where maybe people were thinking it was primarily for girls, because I think it was sort of initially stressed as preventing cervical cancer, but yeah, there’s definitely a big push now for boys to get it.
Theme 2: Agency in adolescence and gauging their adolescent child’s intent for sexual activity	
Parent, age 48 Adolescent, age 15 Vaccination: Tdap Meningococcal	*[Allows adolescent more agency]* Right. And he said, “You know, there’s always cons to everything. I mean, if you take a Tylenol, you know, I mean – you could come up with something.” So our kids have been – we’ve tried to educate them to make their own decisions with that, so unless we have the better education, I don’t see my mind changing.
Parent, age 44 Adolescent, age 14 Vaccinations: 1–2 HPV shots, did not complete by follow-up Tdap Meningococcal Influenza	*[Allows adolescent more agency, gauging adolescent’s intent for sexual activity]* We had a discussion about it and again, I told her the risks as I was aware of them. And we discussed it with her provider a couple different times. I think, too, part of it – I told her what my thought on the timeframe was and she concurred with that. I said if you really want it, if you think that you’re going to be doing things that could put you at risk sooner, then we’ll start sooner.
Parent, age 41 Adolescent, age 12 Vaccinations: 1–2 HPV shots, did not complete by follow-up Tdap Meningococcal	*[Allows adolescent less agency]* I didn’t [talk to my child about getting the vaccine]…I didn’t really think it was something that he should decide.
Parent, age 43 Adolescent, age 11 Vaccinations: HPV (complete) Tdap Meningococcal Influenza	*[Allows adolescent less agency]* I did [talk to my child about the vaccine] a little bit, but just to let her know that she was gonna have it done and what it prevented, but it wasn’t like she had an option.
Theme 3: The credibility of vaccine information sources	
Parent, age 46 Adolescent, age 12 Vaccinations: None	*[Doubtful about credibility of vaccine information sources]* And, you know that they’ll [physicians will] get some perks. That’s life, from pharmaceuticals to doctors, doctors to pharma – probably the first way. But, you just have to hope that people do things for the right reason.
Parent, age 36 Adolescent, age 13 Vaccinations: Tdap Meningococcal	*[Doubtful about credibility of vaccine information sources]* Yes I understand some of these scientists come up with these things and “Hey look, we just discovered this and we can do this.” Okay, yeah and they want the actual positive outcomes for it. And then of course you do have the drug companies, I mean I’m sorry that’s their business and they’re out there to make money. And quite frankly I mean I’m sorry you’re hearing more and more of it on the news…”
Parent, age 48 Adolescent, age 15 Vaccinations: 1–2 HPV shots, did not complete by follow-up Influenza	*[Trusting of vaccine information sources]* I’m just a black or white person. I’m also not a person who has time to do a ton of reading on this stuff. I do more like a little bit of reading and a little bit of talking around and a lot of trusting. Like who is telling me the information, where the sources are where I’m getting my information from. So it’s not like I just go into something blindly. But I think it’s probably maybe it has to do with trusting your practitioner somewhat because that would be the source that gives the information from the very beginning.

### Perceived likelihood of the HPV vaccine preventing cancer

Most parents recognized cancer prevention as a reported benefit of HPV vaccination, but this was not universally accepted. In some cases, cancer prevention was overshadowed by the link of the vaccine to a sexually transmitted infection (STI) among parents of vaccinated and non-vaccinated adolescents. One 44 year old mother whose child (14 years) received Tdap, meningococcal conjugate, and at least one HPV vaccine dose, said,

At first I thought it was interesting that anything that can help prevent any type of cancer is a good thing. But then we started hearing a lot about that it hurt, kids passing out when they had the vaccine and because it was protecting against something that’s sexually transmitted. At the kids’ age my children were, it was kind of like no, we’re not going to go there, yet.

While she absorbed the vaccine’s cancer prevention messaging and eventually did initiate the HPV vaccine, the tie of the vaccine to an STI (and perhaps the perceived reactogenicity of the vaccine) initially overshadowed its ability to prevent cancer in her decision-making.

Other parents were less persuaded by the cancer prevention messaging because they perceived the cancers the vaccine helps prevent as too rare. One such mother aged 42 years, whose 12-year old received Tdap, meningococcal conjugate vaccine, and began (but did not complete) the HPV vaccine series, reported,

Yeah, probably more so the STD [sexually transmitted disease] avenue than the cancer. It doesn’t seem like a really common cancer. I think is it greater in males that aren’t circumcised? I wasn’t too concerned about that but the STD stuff made sense to me so then I – that’s why I did it.

While the mother ultimately made the decision to vaccinate her son, she was more swayed by messaging around STDs, although she was not more specific about her perception of this benefit. One 63 year old parent who declined the HPV vaccine for her child, said quite simply, “Scientific fact is not 100 percent proven to be safe [presumably regarding vaccines]. … But not engaging [in sexual activity] is 100 percent [safe, presumably regarding STD-related cancer risk]. I would recommend those that have that [sexual] activity, expose themselves to the STD, those I probably recommend [the HPV vaccine], but not my relative or my family. Not my family.” One mother, 36 years old, went so far as to see the HPV vaccine’s particular cancer prevention properties as a negative. In the case of a mother whose child received Tdap and meningococcal conjugate vaccines only:

In my case, personally, well cervical cancer runs in the family and there’s forms of cervical cancer that are genetic. And on my dad’s side of the family, it’s the genetic kind. So, some HPV vaccine … in my opinion, it’s not gonna prevent that form of cervical cancer. And then it can cause a false sense of security like, ‘Oh well, I don’t have the money for my Pap smear, so I don’t have to go and get it now because I have the HPV vaccine.’ Or, ‘I got the vaccine, so I’m not gonna get it.’ I feel you have more of a benefit of just getting your yearly Pap smears and you can prevent it and you can catch it early enough that it can be treatable.

While she believed the vaccine to be generally effective on most types of cervical cancer, she also perceived this purported benefit as a potential harm because it may deter women from regular pap exams and result in a net increase in the risk of harm. Another 34 year old mother took a particularly hardline view. With the exception of a prior seasonal influenza vaccine, her 11 year old adolescent child did not receive any of the recommended adolescent vaccines:

I don’t see the need for this HPV [vaccine]. I had a friend at school that was a little more promiscuous, and she ended up having abnormal pap smears and it’s like, at that age, your bodies are still developing and may be more vulnerable to different kinds of viruses and things. I guess I wanna teach [my children] that there’s consequences for actions and this kind of is like, well, that’s not a worry anymore because genital warts and things. ‘I got vaccinated’ so that’s one less reason or something for them to consider. I’m not that naïve to say that everybody’s going to be celibate through high school and after that, but I don’t want them to just check one thing off the list like, ‘I don’t have to worry about that.’ If you knew there was [sic] no cops, would you speed? If there was [sic] no consequences?

She expressed a general anti-vaccine attitude that actualized in refusing most vaccinations, but she presented the threat of HPV as a tool to persuade her children away from unsafe sexual activity. These quotes illustrate the challenge of a universally acceptable/motivating message to promote the HPV vaccine.

### Agency and adolescence and gauging their adolescent child’s intent for sexual activity

As the HPV vaccine is emphasized for 11 and 12 year old adolescents, decisions for or against vaccination occur during a particularly liminal stage in adolescent development. Some parents explicitly acknowledged their sole decision making authority for adolescent vaccinations, while others clearly sought input from their adolescent child. The range of agency parents allowed their children independent of the age of the child highlights the difficulties and ambiguities parents face at this stage in preparing their children for adulthood and independent decision-making. Many parents who allowed their children input in the decision to get the HPV vaccine presented an interesting sub-theme and seemed to consider such moments as ‘tests’ to gauge their child’s intent for sexual activity. This sub-theme seemed to occur more among parents of younger adolescents between ages 11 and 15 (see Table 3). Parents recounted their child refusing the vaccine as affirmations that they were not planning to be sexually active. One 36 year old mother, whose 13 year old child only received Tdap and meningococcal vaccinations, sat up straight and proclaimed about recent conversations with her daughter:

- *Interviewee:* She said, ‘I know my viewpoints and maybe when I get older I might change. If I become sexually active then maybe I’ll change my mind. But right now, know who you’re sleeping with, protect yourself, and that’s gonna be your best frontline against protecting against the HPV.’ … she goes, “that’s your best way to protect and you use protection because once, as long as you’re using protection your risk is gonna be so minimal anyway.’ She says that she has no worries about it. She goes, and come the time when she’s not gonna use protection…‘I’m gonna be married, so …’ She said she’s not worried about it. And so, I just told her, I’m like, ‘Well, it’s fine because like I said, I’m not gonna make you get it; it’s your choice. If you ever change your mind before you’re eighteen, let me know but otherwise, it’s your body; you make the decision yourself’ … I’ve raised her to be independent and make her own choices.
- *Interviewer:* So, what would you have done if, say, your daughter said, ‘Mom, I really wanna get this vaccine?’
- *Interviewee:* I would ask her, ‘why?’ What information would lead her to think that she wants it or needs it?

Regardless of whether this conversation happened the way the mother recounted it, her lasting impression of the exchange is what is at stake and certain elements of her conversation are generalizable across interviews. This mother appealed to her daughter’s autonomy (“It’s your body; you make the decision yourself”) to open up a conversation about her daughter’s attitudes towards sex (“and come the time when she’s not gonna use protection…‘I’m gonna be married.’). Also, the mother often melds her voice with the words of her child, as though they speak as one individual while at the same time praising her daughter’s independence and ability to make her own decisions. This linguistic technique underscores how as adolescents seek more autonomy, many parents appeared to struggle with when to cede parental authority, particularly regarding the salient topic of sexual decisions. Thus the HPV vaccine decision can be a point where parents seek to clarify their adolescent’s attitudes and/or intent for sexual decision-making. Put more plainly, it was an affirming moment for many parents to hear their child echo their desire to remain a virgin until marriage. This seemed to obviate the (perhaps feared) necessity to reinforce the importance of sexual purity if their child requests the vaccine (i.e., “I would ask her, ‘why?’. What information would lead her to think that she wants or needs it?”).

### The credibility of HPV vaccine information sources

Not all information sources regarding the HPV vaccine were seen as equally useful. Parents of vaccinated and non-vaccinated adolescents alike distrusted pharmaceutical companies. One 39 year old father, whose 11 year old adolescent received all the recommended adolescent vaccines said,

I’m somewhat cynical that it could also happen that – oh, vaccines, they’re a great money-maker. Everybody has to take them. Let’s prove that this is good, and then tell everybody to go get pumped full of it. And it’ll be years down the road, and they could potentially never prove whether this vaccine caused something, or didn’t prevent something. Meanwhile, we still have all the money … that the deck is stacked against you by the big companies, and the government, and you’re so little – you have little-to-no chance to know if you’re really following what’s good information or not.

Interestingly, a 48 year old parent whose 15 year old adolescent had not received any recommended adolescent vaccinations, echoed similar concerns to justify different decisions:

- *Interviewee:* Well, we recommend a lot of things that down the road, aren’t positive for us. So yeah.
- *Interviewer*: Why do you think that happens? Why do you think they’re recommended in the first place?
- *Interviewee:* I think that they see some value in some study, which is usually put out by the drug makers, themselves, you know?

While pharmaceutical companies were distrusted, almost all participants expressed a high degree of trust in their doctor’s advice. One father of three sons when probed over how much he pushed his children to get the vaccine stated, “I don’t think we necessarily pushed it, but, when we were advised by the pediatrician to get it, we went ahead and did it.” In other words, the father did not express a strong inclination to vaccinating his adolescents against HPV, but the pediatrician’s recommendation was the main driver of that decision.

The interplay between the pharmaceutical industry and providers was also apparent. Again, parents did not see information from pharmaceutical companies as persuasive, but recognize that it may impact their provider’s advice that they give parents. One mother of four children, whose youngest (11 year old) adolescent had not received any of the recommended adolescent vaccinations, described the intimidation many parents feel in evaluating information on medications:

It’s hard to research, to find a—you look it up online and you have to find a reputable source. Okay, you go under Gardasil… And you know they’re not per se going to persuade you not to have it. All the information is going to presumably be, ‘you should get it’ because it’s their product; they’re selling their product. And sometimes I feel the medical field—there’s so much to know that they just—they get the information from like the [pharmaceutical sales] reps. But what is their focus? Is to spread or promote their business? It’s hard to find a real neutral source on that.

When asked if she trusts the doctor when s/he recommends the vaccine, she responded,

I trust them in the fact of it’s kind of a cookie cutter—you look at statistics. X amount are sexually active by this point, so you wanna get it before, and I don’t wanna think of my child as just a statistic, as in ‘well, that’s bound to happen. She’s gonna get it, so now you need to prevent it’, when it [sexual activity] is a personal choice [versus] a personal action like whooping cough. You’re in school, someone coughs on you, that was not your fault.

This mother was unconvinced by the logic from health statistics related to HPV, and seemed to put more value in her child’s sexual choices as a means to avoid HPV infection. The physician was not seen as necessarily biased by the pharmaceutical company, but there seemed to be a desire for more personalized advice that balanced HPV health statistics with her family’s risk factors.

## Discussion

We conducted semi-structured interviews with north-central Wisconsin parents in an attempt to better understand factors that influenced changes in their attitudes about the HPV vaccine and related decisions. Interviewed parents seemed to be in the midst of HPV vaccine decision-making, as nearly three-fourths of their adolescent children had at least one HPV vaccine dose at the time of the study interview. The observed themes in this study did not aptly relate to changes in HPV vaccine attitudes as originally intended, however, as the survey-measured shifts in HPV vaccine attitudes ‘on paper’ were unnoticeably modest or otherwise rarely salient enough for parents to comment on during their interviews. Reasons for this imperceptibility in CHIAS change scores were not directly probed as part of the interviews, but three main themes emerged that were antecedent to parents’ HPV vaccine attitudes, including: (1) the perceived likelihood of the HPV vaccine preventing cancer, (2) gauging their adolescent child’s intent for sexual activity, and (3) the credibility of HPV vaccine information sources.

The cancer prevention message most discernably promoted with HPV vaccination was widely recognized across interviewees, but was not universally convincing as some parents considered the likelihood of HPV vaccine-preventable cancer remote in their adolescent child (overall and in light of regular cancer screenings such as pap smears). This theme was generally consistent with at least two prior studies as well, where parents voiced limited concern about their adolescents’ susceptibility to HPV infection [9, 13]. One parent who refused the HPV vaccine for her daughter also considered the cervical cancer risk a worthwhile deterrent to unsafe sexual practices. This suggests that general messaging around the HPV vaccine’s effectiveness regarding cancer prevention may need richer context for some parents, as it may be diluted by perceived low susceptibility to vaccine-preventable cancers [8, 19]. General beliefs about vaccines, as well as attitudes towards sex and maturity in particular, can collude in unexpected ways to foster ambivalence or antipathy toward the HPV vaccine.

This balance between risk and responsibility was also apparent in the second theme, where parents seemed to be concerned about the appropriateness of the timing of the HPV vaccine, as has been observed by others [20] and again intimated in another parent interview study [13]. A novel insight, however, was that parents often positioned the HPV vaccine decision as a means to gauge their adolescent child’s intent for sexual activity. Other studies have suggested adolescents are generally passive about HPV vaccine decisions, at least until older ages [13, 21, 22], but there was ambiguity regarding how much autonomy adolescents actually have in this regard. Some parents seemed to relish their adolescents’ autonomy on HPV vaccine decisions so long as they aligned with their own views. There were indications that parental enthusiasm for their adolescent’s autonomy might wane if their views on the HPV vaccine, which could be viewed as a harbinger of sexual activity, were opposing.

It was clear from our interviews, as in other prior investigations [7, 9, 13, 23], that parents often look to their healthcare provider for advice about the HPV vaccine and this informs their attitudes. But this educational burden may be more nuanced than previously appreciated. In addition to their doctor’s advice, parents in our study usually considered multiple information sources about the HPV vaccine. They expressed more pointed skepticism, however, of pamphlets received in the mail or delivered in the clinic, often saying they quickly disregarded them in suspicion they were provided by the pharmaceutical industry. Parents looked to their physician to filter the various, sometimes conflicting, messages about the HPV vaccine and provide bottom-line guidance. Clearly delivered cancer prevention facts about the HPV vaccine may help some parents, but other parents felt swayed by their doctor’s invitation to consider things they had not previously thought about, such as the sexual history of their child’s future life partner(s) (vs. the sanctity of their own child’s sexual decisions). Such comments did not rise to the level of a common theme, but suggest that other personalized or family-centered communication/message delivery strategies (to help some parents gain fuller appreciation for the risks of HPV infection) are an important area of future study.

This study was strengthened by the in-depth data collection methods, which included information on participants’ prior vaccination history and the defined source population. There were several limitations as well. Adolescents and physicians are stakeholders in HPV vaccine decisions too, but were not directly included in our study interviews. We interviewed predominately White mothers from a relatively homogenous area of the U.S. And though there were several participants with negative views about the HPV vaccine, the sample may have held somewhat more favorable attitudes in general toward vaccines given the high levels of education and majority employment in healthcare and education sectors. Similarly, this study was conducted as part of a broader effort to increase HPV vaccine coverage within select MCHS centers. Thus, interviewed parents may have been more engaged in the topic of HPV vaccination relative to others, particularly considering they also completed two prior surveys on the topic of HPV vaccine attitudes.

Interviews with parents of adolescents revealed several important factors that influenced their HPV vaccine attitudes. These included mixed aspects of cancer prevention and the use of the HPV vaccine discussions to gauge adolescent sexual intent. Also, parents felt a sense of responsibility to educate themselves and their children about the vaccine, particularly looking to their doctor to filter the various information sources on the topic. Future research should examine how to optimize clinical messaging strategies that can sway hesitant parents toward more favorable HPV vaccine attitudes, and how such attitudinal shifts might eventually increase HPV vaccine coverage in more diverse patient populations.

## Abbreviations

CHIAS: Carolina HPV Immunization Attitudes and Beliefs Scale
HPV: Human Papillomavirus
MCHS: Marshfield Clinic Health System
STD: Sexually transmitted disease
STI: Sexually transmitted infection
Tdap: Tetanus, diphtheria, and pertussis
